# BNIP3 contributes to cisplatin‐induced apoptosis in ovarian cancer cells

**DOI:** 10.1002/2211-5463.12881

**Published:** 2020-06-27

**Authors:** Jinghui Jia, Xiaoxin Yang, Qing Zhao, Feiquan Ying, E Cai, Si Sun, Xiaoqi He

**Affiliations:** ^1^ Department of Obstetrics and Gynecology Air Force General Hospital, PLA Beijing China; ^2^ Department of Obstetrics and Gynecology Tongji Medical College Union Hospital Huazhong University of Science and Technology Wuhan China; ^3^ Department of Obstetrics and Gynecology Tongren Hospital of WuHan University (Wuhan Third Hospital) Wuhan China; ^4^ Department of Obstetrics and Gynecology Tongji Medical College The Central Hospital of Wuhan Huazhong University of Science and Technology Wuhan China

**Keywords:** apoptosis, BNIP3, cisplatin, cisplatin sensitization, ovarian cancer

## Abstract

BNIP3 is a proapoptotic protein that mediates apoptosis, necrosis and autophagy. However, the involvement of BNIP3 in cisplatin‐induced apoptosis in ovarian cancer is not clear. In this study, we examined the role of BNIP3 in ovarian cancer during cisplatin treatment and its correlation with clinical outcomes. We first measured cisplatin cytotoxicity and BNIP3 levels before and after cisplatin exposure for ovarian cancer cell lines A2780, SKOV3, OVCAR4, OV2008, ES2 and HO8910. BNIP3 was observed to be differentially expressed in these cell lines, and cisplatin induced a significant increase in BNIP3 levels in A2780 and OVCAR4. BNIP3 knockdown with siRNA in A2780 and OVCAR4 significantly reduced cisplatin cytotoxicity in these two cell lines and alleviated cisplatin‐induced apoptosis. We searched the online databases Gene Expression Omnibus and The Cancer Genome Atlas to analyze the correlation between BNIP3 level and overall survival and progression‐free survival in patients with ovarian cancer. Pooled analyses showed that higher BNIP3 level was correlated with poorer overall survival (95% confidence intervals; hazard ratio = 1.18, 1.04–1.34; *P* = 0.013) and progression‐free survival (95% confidence intervals; hazard ratio = 1.26, 1.10–1.43; *P* = 0.00049). However, the results of individual datasets and stratification analyses of histology, FIGO (Federation Internationale de Gynecolgie et d’Obstetrique) stage, chemotherapy regimen and P53 mutation status varied. These findings indicate that cisplatin‐induced apoptosis is dependent on BNIP3 level in ovarian cancer cell lines. Targeting BNIP3 may therefore be a potential way of restoring cisplatin sensitivity.

## Graphical abstract

BNIP3 is up‐regulated by cisplatin in ovarian cancer cells and contributes to cisplatin‐induced cellular apoptosis in a caspase‐dependent manner.

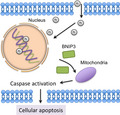

**Abbreviations**
FIGO: Federation Internationale de Gynecolgie et d'Obstetrique
HR: hazard ratio
MTT: 3‐(4,5‐dimethylthiazol‐2‐yl)‐2,5‐diphenyl‐tetrazolium bromide
NC: cell lines treated with saline
OS: overall survival
PFS: progression‐free survival
polyAb: polyclonal antibody
qRT‐PCR: quantitative RT‐PCR
SD: standard deviation
si‐BNIP3: cell lines transfected with BNIP3 siRNA
si‐NC: cell lines transfected with NC siRNA
TCGA: The Cancer Genome Atlas

Ovarian cancer is the most lethal gynecological malignancy. The asymptomatic early‐stage disease usually advances to FIGO (Federation Internationale de Gynecolgie et d’Obstetrique) stage III‐IV drastically almost unnoticed, leading to a prevalence of late diagnosis and poor outcome [1, 2]. The standard treatment for ovarian cancer with FIGO stage II‐IV is tumor‐debulking surgery followed by platinum‐based chemotherapy [3]. Around 70% of patients gradually develop platinum resistance, subsequent chemotherapy failure and recurrence [4]. The 5‐year survival rate of patients with ovarian cancer is only <30% [5]. Improving the overall survival (OS) of patients with ovarian cancer remains the most challenging task.

As the two most commonly used anticancer chemotherapeutic agents in treatment of multiple solid malignancies, including ovarian cancer, cisplatin and carboplatin share a similar cytotoxic mechanism [6]. The binding of platinum complex to DNA leads to DNA abducts formation and DNA configuration alteration, which causes cell‐cycle arrest and the release of both proapoptotic and prosurvival signals. The ultimate fate of the assaulted cells depends on the duration and intensity of the activated signals, the interaction of both proapoptotic and prosurvival pathways, and the extent of DNA damage [7]. The apoptosis of ovarian cancer cells triggered by cisplatin could be either caspase dependent or independent [8]. Bax/Bcl2 and Fas/FasL contribute to cisplatin‐induced apoptosis through caspase‐9/caspase‐3 and caspase‐8/caspase‐3 pathways, respectively [9], whereas the AKT/ERK‐FOXO3a pathway has a negative effect on caspase‐independent apoptosis induced by cisplatin [10].

BNIP3 is a BH3‐only protein involved in cell death through mediating apoptosis, necrosis and autophagy [11]. As a member of the Bcl‐2 family, it acts as a proapoptotic protein that interacts with Bcl‐2 and activates the downstream Bax/Bak caspase‐dependent apoptotic pathway [12]. In cardiomyocytes, BNIP3 mediates necrosis caused by ischemia–reperfusion through the Ripk3–JNK–Bnip3–mitochondria cascade [13]. In malignant glioma cells, BNIP3 causes mitochondria depolarization and autophagic cell death in reaction to ceramide [14]. As a key mediator protein in autophagy, BNIP3 is responsive to hypoxia via the carboxyl‐terminal transmembrane domain. Subsequent interaction of BNIP3 with LC3 through LC3‐interacting regions promotes hypoxia‐induced autophagy [15].

The aforementioned diverse effects of BNIP3 in cell death led to intricate roles of BNIP3 in cancer. The basic level of BNIP3 in tumors was tissue specific. BNIP3 was highly expressed in breast cancer, lung cancer, glioma and cervical cancer. BNIP3 was epigenetically silenced in pancreatic cancer, colorectal cancer, gastric cancer, leukemia and lymphoma [16]. Induction of BNIP3 by HIP‐1 under hypoxia found in various solid tumors was regarded as one of the factors promoting tumor aggressiveness, increasing resistance to therapy and worsening overall patient survival [17]. In ovarian cancer, BNIP3 was reported to influence the proliferation and migration of ovarian cancer cells [18]. In addition, the truncated form of BNIP3 lacking the transmembrane domain abolished the mitochondria‐dependent cellular apoptosis induced by BNIP3 in ovarian cancer cells [19]. Evidence suggested that BNIP3 also reacted to chemotherapy agents, such as paclitaxel [20]. However, the effect of BNIP3 regarding cell death in reaction to cisplatin in ovarian cancer is not clear.

Therefore, in this study, we aim to evaluate the function of BNIP3 in ovarian cancer during cisplatin treatment and its correlation with clinical outcomes.

## Materials and methods

### Cell culture

Human ovarian cancer cell lines A2780, SKOV3, OV2008, OVCAR4, HO8910 and ES2 were purchased from the China Center for Type Culture Collection (CCTCC, Wuhan, China). A2780 and ES2 were cultured with 1640 medium supplemented with 10% FBS, whereas SKOV3, OV2008, OVCAR4 and HO8910 were cultured with Dulbecco’s modified Eagle’s medium/F12 medium supplemented with 10% FBS (Gibco; Thermo Fisher Scientific, Inc., Waltham, MA, USA). All media were added to 1% penicillin and streptomycin. All cells were maintained at 37 °C with 5% CO_2_ in a humidified incubator.

### 3‐(4,5‐Dimethylthiazol‐2‐yl)‐2,5‐diphenyl‐tetrazolium bromide assay

The cytotoxic effect of cisplatin to ovarian cancer cell lines A2780, SKOV3, OV2008, OVCAR4, HO8910 and ES2 and siRNA‐transfected cell lines A2780siBNIP3/A2780siNC and OVCAR4siBNIP3/OVCAR4siNC was assessed using the 3‐(4,5‐dimethylthiazol‐2‐yl)‐2,5‐diphenyl‐tetrazolium bromide (MTT) assay. Cells were seeded in 96‐well plates (Corning Life Sciences, Corning, NY, USA) at a density of 5000 cells per well. After attachment, the cells were exposed to different concentrations of cisplatin for 48 h (0, 2.5, 5, 10, 20, 40, 60, 80 and 100 μm) and followed by 4 h‐incubation at 37 °C in 5 mg·mL^−1^ medium diluted with MTT reagent (Sigma‐Aldrich, Merck KGaA, Darmstadt, Germany) [21, 22]. The medium was removed, and 100 μL pure dimethyl sulfoxide was added to each well overnight for consecutive absorbance (*A*) measurement at 490 nm wavelength using a plate reader (iMark‐10601; Bio‐Rad Laboratories, Inc., Hercules, CA, USA). Cell survival curves were generated while reads from wells with no cells served as blank controls and reads from wells with pure medium served as negative controls. All experiments were performed in triplicate.

### Western blot analysis

Total cellular proteins were extracted using a protein extraction kit (Beyotime Institute of Biotechnology, Haimen, China). The cellular lysates were subjected to protein quantification by bicinchoninic acid assay (Beyotime Institute of Biotechnology) according to the manufacturer’s protocol. Fifty micrograms of each sample was loaded for the blotting process and then transferred to poly(vinylidene difluoride) membranes. The membranes were blocked with 5% nonfat milk at room temperature for 1 h followed by incubation of the primary antibodies at 4° C overnight. The primary antibodies used were Anti‐BNIP3 Rabbit serum, Anti‐PMS2 Rabbit serum (Abcam, Cambridge, MA, USA), anti‐(cleaved caspase‐3) rabbit serum, anti‐[Poly ADP‐ribose polymerase (PARP) cleavage rabbit] serum, Anti‐LC3B Rabbit serum (Cell Signaling Technology, Inc., Danvers, MA, USA) and Anti‐β‐actin Mouse serum (Epitomics Inc., Burlingame, CA, USA). Then the membranes were washed three times using Tris‐Buffered Saline Tween‐20 buffer before incubation in the secondary antibodies at room temperature for 1 h. The secondary antibodies used were horseradish peroxidase‐conjugated goat anti‐(rabbit Ig) and goat anti‐(mouse Ig) (both from Santa Cruz Biotechnology, Inc., Dallas, TX, USA). Before visualization, the membranes were soaked in an enhanced chemiluminescence system (Beyotime Institute of Biotechnology) and exposed with Molecular Imager Chemi‐Doc XRS+ system (Bio‐Rad Laboratories, Inc.). The bands were quantified using quantity one v4.62 software (Bio‐Rad Laboratories, Inc.). All experiments were performed in triplicate.

### Quantitative RT‐PCR

Quantitative RT‐PCR (qRT‐PCR) assays were carried out as previously described. In brief, total RNAs were extracted from the harvested cells using TRIzol (Invitrogen, Carlsbad, CA, USA) according to the manufacturer’s instructions. Reverse transcription was performed using a Reverse Transcription Kit (Toyobo, Osaka, Japan). PCRs were performed using an Applied Biosystem StepOne Plus PCR system (ABI, Foster City, CA, USA) with SYBR Green Real‐time PCR Master Mix (TaKaRa, Otsu, Japan). The primers were designed based on National Center for Biotechnology Information (NCBI) reference sequences and synthesized by Invitrogen (Suzhou, China). The primers used were as follows: BNIP3, forward 5′‐GCCCACCTCGCTCGCAGACAC‐3′, reverse 5′‐CAATCCGATGGCCAGCAAATGAGA‐3′; and β‐actin, forward 5′‐CAGAGCCTCGCCTTTGCC‐3′, reverse 5′‐GTCGCCCACATAGGAATC‐3′. All primers were assessed by a standard curve, ensuring the amplification efficiencies to be approximately 100%. BNIP3 expression was normalized to β‐actin and calculated using the $2^{-\Delta \Delta C_{t}}$ method. All experiments were performed in triplicate.

### Cell transfection

Small interfering BNIP3‐specific siRNA and a scrambled control siRNA were synthesized by Univ‐bio (Shanghai, China). The sequence for siBNIP3 was 5′‐AAGGAACACGAGCGUCAUGAAdTdT‐3′. The siRNA transient transfections were performed using Lipofectamine 2000 (Invitrogen) according to the manufacturer’s instruction. The transfected cells were exposed to puromycin selection for 3 days prior to further experiments.

### Clonogenic assay

Cells were seeded to six‐well plates at a concentration of 500 cells per well. When cells were attached, they were exposed to different concentrations of cisplatin for 48 h; then media were changed with fresh media, and culturing continued for another 10–14 days until visible colonies were seen. The colonies were fixed using 4% formaldehyde for 30 min and stained with crystal violet for at least 1 h. The plates were dried and photographed for colony counting. All experiments were performed in triplicate.

### Flow cytometry for apoptosis

A2780siBINP3/A2780siNC and OVCAR4siBNIP3/OVCAR4siNC cells were seeded in six‐well plates at a concentration of 1 × 10^5^ cells per well. When grown to 90% of confluence, the cells were exposed to cisplatin (6 μm for A2780 and 8 μm for OVCAR4) for 48 h. The cells were harvested by trypsin without EDTA, washed twice with icy PBS and went on for apoptosis detection using a FITC Annexin V Apoptosis Detection kit (catalog no. 556547; BD Biosciences, San Jose, CA, USA) according to the manufacturer’s protocol. The cells were analyzed on a flow cytometer (FACSCalibur; BD Biosciences). The data were analyzed by cellquest™ analysis program software, version 5.1 (BD Biosciences). All experiments were performed in triplicate.

### Bioinformatics analysis

RNA sequencing data of 10 datasets [The Cancer Genome Atlas (TCGA), GSE9891, GSE26712, GSE26193, GSE63885, GSE65986, GSE18520, GSE30161, GSE3149, GSE14764] were downloaded from the TCGA (https://tcga‐data.nci.nih.gov/tcga/) and Gene Expression Omnibus websites (http://www.ncbi.nlm.nih.gov/geo/). An open free online survival analysis website for the Kaplan–Meier plotter (http://kmplot.com/analysis/) was used to analyze the prognosis significance of BNIP3 in patients with ovarian cancer. The transcript for BNIP3 measured in each dataset was 201849_at with the best probe set. Median expression of BNIP3 within each dataset was selected as the cutoff point dividing BNIP3 expression as high or low.

### Statistical analysis

Statistical analysis was performed using spss 17.0 statistics software (IBM, New York, NY, USA) and graphpad prism 5.0 software (GraphPad Software, San Diego, CA, USA). The statistical significance of differences between two groups was analyzed using two‐tailed Student’s *t*‐test. The statistical significance of differences among three or more groups was analyzed using one‐way ANOVA. Kaplan–Meier and log rank tests were used to evaluate OS and progression‐free survival (PFS). All data are expressed as the mean ± standard deviation (SD) (*n* ≥ 3).

## Results

### BNIP3 level in ovarian cancer cell lines

**Fig. 1:**
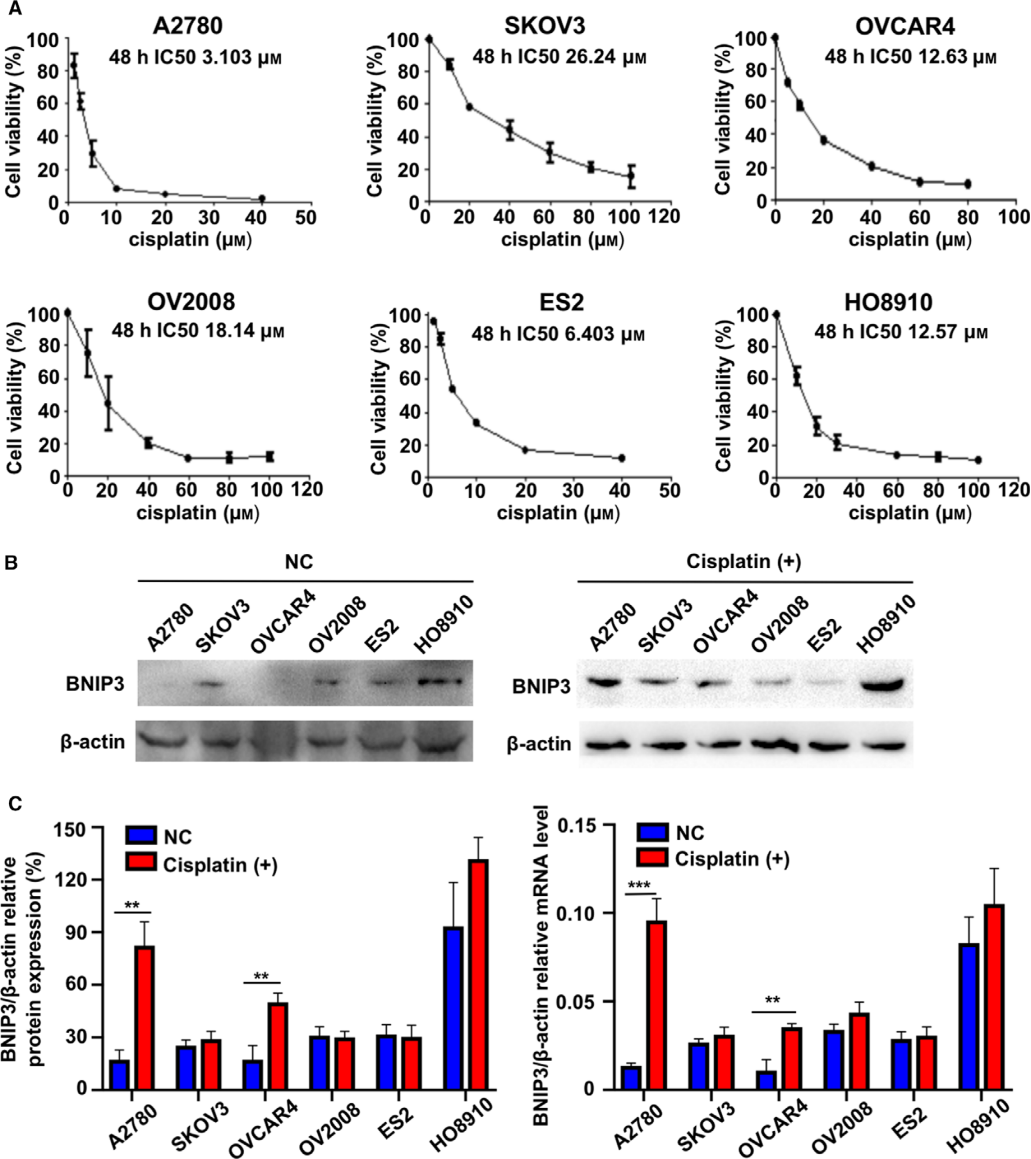
BNIP3 level in ovarian cancer cell lines. (A) The cell viability of ovarian cancer cell lines measured by MTT 48 h after exposure to indicated concentrations of cisplatin. The IC50 of the ovarian cells were A2780 3.103 μm, OV2008 18.14 μm, SKOV3 26.24 μm, OVCAR4 12.63 μm, ES2 6.403 μm and HO8910 12.57 μm. (B–D) BNIP3 level before and after cisplatin exposure measured by western blotting (B) and by qRT‐PCR (C, D) in A2780, SKOV3, OVCAR4, OV2008, ES2 and HO8910 cells. Data were presented as mean ± SD, *N* = 3. **P < 0.01, ***P < 0.001, Student’s *t*‐test.

First, the cytotoxicity of cisplatin to ovarian cancer cell lines A2780, SKOV3, OVCAR4, OV2008, ES2 and HO8910 was determined using the MTT test. The general platinum cytotoxicity was estimated by the average the half maximal inhibitory concentration (IC50) of all cell lines included. The cell lines with an IC50 below the average level were considered more sensitive. As shown in Fig. 1A, A2780 (IC50, 3.103 μm), OVCAR4 (IC50, 12.63 μm), ES2 (IC50, 6.403 μm) and HO8910 (IC50, 12.57 μm) cells were relatively more sensitive to cisplatin treatment than the rest of the cell lines. Next, we evaluated the BNIP3 level in all of the earlier cell lines before and after cisplatin treatment. We found that BNIP3 is generally expressed in all six cell lines with lower basal level in A2780 and OVCAR4 and higher level in HO8910 (Fig. 1B). With cisplatin treatment, the protein level of BNIP3 in A2780 and OVCAR4 increased, respectively, with corresponding mRNA increase (Fig. 1B–D).

### BNIP3 and cisplatin cytotoxicity in ovarian cancer cell lines

**Fig. 2:**
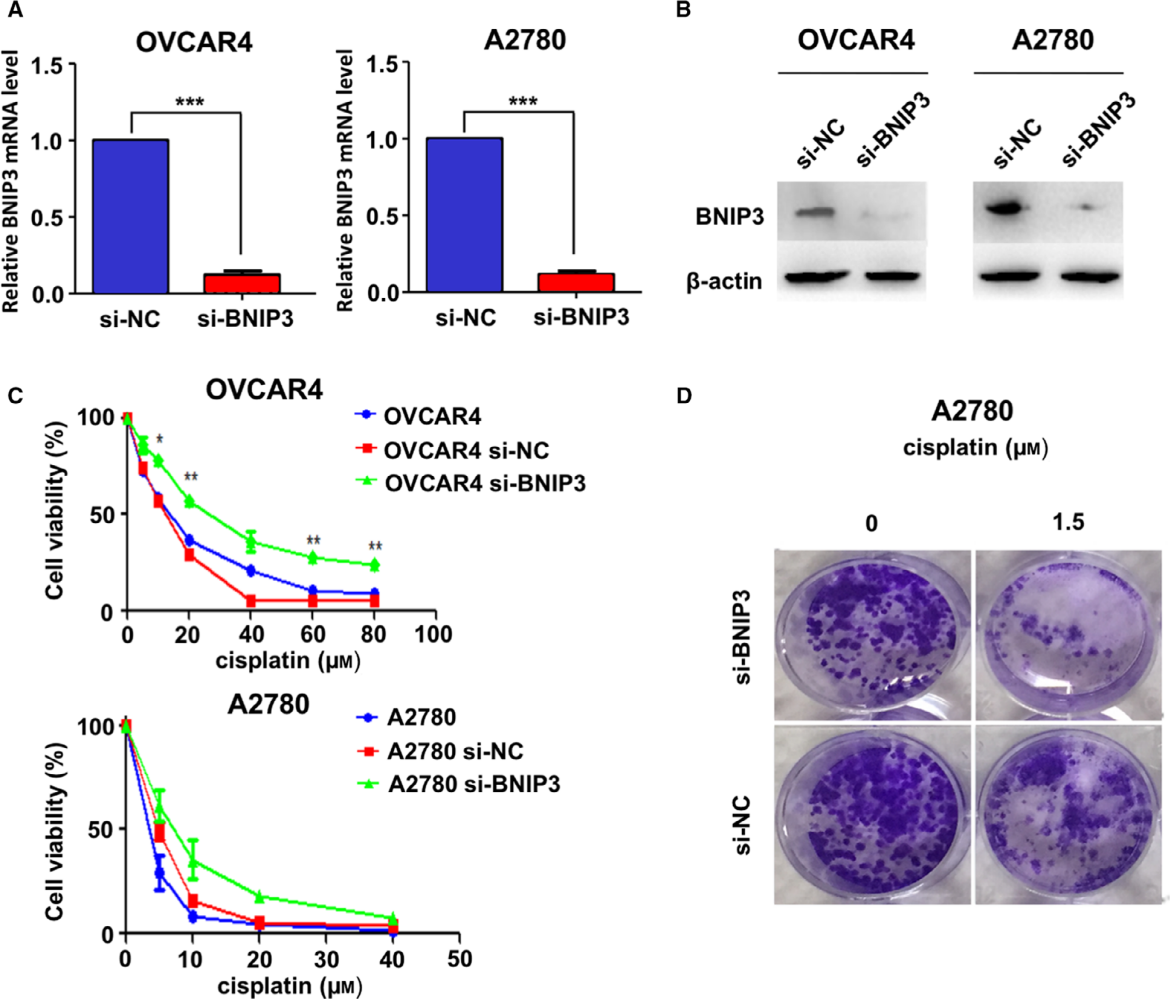
BNIP3 level is correlated with cisplatin cytotoxicity in ovarian cancer cell lines. (A, B) Relative BNIP3 mRNA level measured by qRT‐PCR (A) and BNIP3 protein level by western blotting (B) in A2780 and OVCAR4 cells after BNIP3 knockdown. (C, D) Cell viability was measured by MTT (C) and clonogenic ability was measured by clonogenic assay (D) in A2780 and OVCAR4 after BNIP3 knockdown. Data were presented as mean ± SD, *N* = 3. *P < 0.05, **P < 0.01, ***P < 0.001, Student’s *t*‐test.

To investigate whether BNIP3 level is correlated with cellular cisplatin cytotoxicity, we knocked down BNIP3 expression in relatively platinum‐sensitive ovarian cancer cell lines to determine whether any concurrent cisplatin sensitivity variation occurs. Because A2780 and OVCAR4 were of better cisplatin sensitivity, more significant BNIP3 increase in reaction to cisplatin treatment and better transfection efficiency, we proceeded the subsequent experiments using these two cell lines. The protein and mRNA levels of BNIP3 of A2780 and OVCAR4 are down‐regulated in A2780si‐BNIP3 and OVCAR4si‐BNIP3 (Fig. 2A,B). The decrease in BNIP3 leads to a declined cellular response to cisplatin measured by MTT assay and clonogenic assay (Fig. 2C,D).

### Cisplatin‐induced cellular apoptosis was dependent on BNIP3

**Fig. 3:**
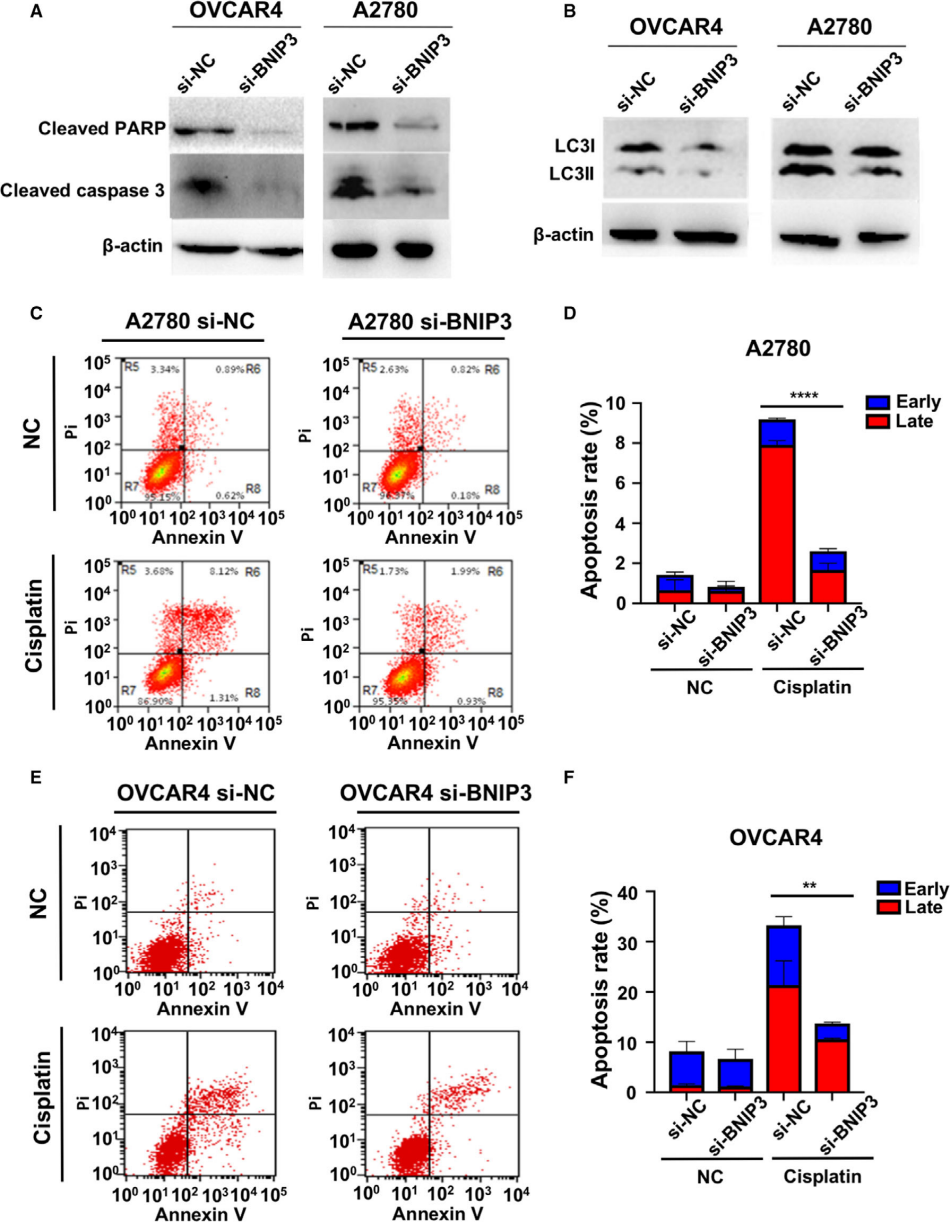
Cisplatin‐induced cellular apoptosis was dependent on BNIP3. (A, B) Cleaved PARP and caspase‐3 (A) and LC3I/LC3II (B) were measured by western blotting in A2780 and OVCAR4 cells when treated with 6 and 8 μm CDDP for 48 h after BNIP3 knockdown. (C, E) Apoptosis and necrosis in A2780 si‐NC/A2780 si‐BNIP3 (C) and OVCAR4 si‐NC/OVCAR4 si‐BNIP3 (E) cells assessed by flow cytometry following 48 h of 6 and 8 μm cisplatin treatment. (D, F) The apoptotic percentage in A2780 si‐NC/A2780 si‐BNIP3 (D) and OVCAR4 si‐NC/OVCAR4 si‐BNIP3 (F) cells after treatment of cisplatin. Data were presented as mean ± SD, *N* = 3. **P < 0.01, ****P < 0.0001, Student’s *t*‐test.

Because BNIP3 correlated with cisplatin cytotoxicity in ovarian cancer cells, we hypothesized that cisplatin‐induced cellular apoptosis was to some extent dependent on BNIP3 level. To determine the cellular status after cisplatin exposure, we measured the markers of apoptosis, including cleaved PARP and cleaved caspase‐3, as well as the markers of autophagy, including LC3I and LC3II in A2780 and OVCAR4 cells. BNIP3 knockdown led to a significant decrease in platinum‐induced PARP/caspase‐3 cleavage and slight reduction of LC3I/LC3II level (Fig. 3A,B). According to flow cytometry results, cisplatin treatment caused apoptosis in both cell lines transfected with NC siRNA (si‐NC) and cell lines transfected with BNIP3 siRNA (si‐BNIP3) cells of A2780 and OVCAR4. BNIP3 down‐regulation did not bring about much change in apoptotic rate in saline‐treated cells, but significantly alleviated cisplatin‐induced apoptosis in both A2780 and OVCAR4 (Fig. 3C–F and Table 1). Based on the earlier results, we assumed that cisplatin‐induced cellular apoptosis was dependent on BNIP3.

**Table 1:** Apoptosis and necrosis of ovarian cancer cell lines A2780 and OVCAR4 in reaction to cisplatin after BNIP3 regulation. Cisplatin, cell lines treated with cisplatin. *P*‐value, unpaired student *t*‐test. Data are presented as mean ± SD.

Apoptosis/Necrosis	A2780			OVCAR4		
	NC	Cisplatin	*P*	NC	Cisplatin	*P*
si‐NC						
Early apoptosis	0.77 ± 011	1.28 ± 0.05	**0.0040**	6.87 ± 1.63	11.85 ± 0.23	**0.0276**
Late apoptosis	0.67 ± 0.42	7.91 ± 0.18	**<0.0001**	1.46 ± 0.17	21.39 ± 3.93	**0.0020**
Apoptosis	1.43 ± 0.36	9.18 ± 0.18	**<0.0001**	8.15 ± 1.47	33.24 ± 5.32	**0.0030**
Necrosis	2.98 ± 0.26	3.10 ± 0.50	0.7909	0.40 ± 0.29	1.42 ± 0.14	**0.0101**
si‐BNIP3						
Early apoptosis	0.21 ± 0.03	0.93 ± 0.11	**0.0008**	5.49 ± 1.56	3.13 ± 0.23	0.1021
Late apoptosis	0.63 ± 0.08	1.67 ± 0.27	**0.0368**	1.17 ± 0.09	10.58 ± 0.18	**<0.0001**
Apoptosis	0.83 ± 0.12	2.60 ± 0.23	**0.0060**	6.66 ± 1.47	13.71 ± 0.36	**0.0028**
Necrosis	3.00 ± 0.28	2.20 ± 0.35	0.0640	0.64 ± 0.28	0.99 ± 0.07	0.1576

	A2780			OVCAR4		
	si‐NC	si‐BNIP3	*P*	si‐NC	si‐BNIP3	*P*
NC						
Early apoptosis	0.77 ± 011	0.21 ± 0.03	**0.0022**	6.87 ± 1.63	5.49 ± 1.56	0.4951
Late apoptosis	0.67 ± 0.42	0.63 ± 0.08	0.9268	1.46 ± 0.17	1.17 ± 0.09	0.0995
Apoptosis	1.43 ± 0.36	0.83 ± 0.12	0.1946	8.15 ± 1.47	6.66 ± 1.47	0.3700
Necrosis	2.98 ± 0.26	3.00 ± 0.28	0.9437	0.40 ± 0.29	0.64 ± 0.28	0.4365
Cisplatin						
Early apoptosis	1.28 ± 0.05	0.93 ± 0.11	**0.0147**	11.85 ± 0.23	3.13 ± 0.23	**0.0010**
Late apoptosis	7.91 ± 0.18	1.67 ± 0.27	**<0.0001**	21.39 ± 3.93	10.58 ± 0.18	**0.0177**
Apoptosis	9.18 ± 0.18	2.60 ± 0.23	**<0.0001**	33.24 ± 5.32	13.71 ± 0.36	**0.0066**
Necrosis	3.10 ± 0.50	2.20 ± 0.35	0.1085	1.42 ± 0.14	0.99 ± 0.07	**0.0171**

Values in bold represent values with statistical significance.

### BNIP3 level and clinical outcome of patients with ovarian cancer

Because BNIP3 level was significantly related to cisplatin‐induced apoptosis, we wondered whether BNIP3 could act as a biomarker for clinical outcomes in patients with ovarian cancer. We performed the analyses to examine the correlation between BNIP3 level and clinical outcomes such as OS and PFS in patients with ovarian cancer using online datasets. Pooled analyses showed that higher BNIP3 level was correlated with poorer OS [95% confidence intervals (CI); hazard ratio (HR) = 1.18, 1.04–1.34; *P* = 0.013] and PFS (95% CI; HR = 1.26, 1.10–1.43; *P* = 0.00049; Table 2). However, this correlation might vary on account of histology, FIGO stage, chemotherapy regimen and P53 mutation status according to stratification analyses. High BNIP3 level remained correlated with poor OS in patients with serous (OS: 95% CI; HR = 1.23, 1.05–1.43; *P* = 0.0085) and advanced stage (OS: 95% CI; HR = 1.23, 1.06–1.43; *P* = 0.0058) ovarian cancer. Although in patients who received paclitaxel (OS: 95% CI; HR = 0.57, 0.36–0.90; *P* = 0.014; PFS: 95% CI; HR = 0.54, 0.35–0.84; *P* = 0.0053) or topotecan (OS: 95% CI; HR = 0.58, 0.37–0.90; *P* = 0.013) and patients with wild‐type p53 (PFS: 95% CI; HR = 0.47, 0.24–0.93; *P* = 0.027), high BNIP3 level was related with better OS or PFS (Table 2). Then an independent dataset was analyzed separately. GSE9891 (OS: 95% CI; HR = 1.46, 1–2.13; *P* = 0.046) and GSE65986 (PFS: 95% CI; HR = 3.53, 0.99–12.57; *P* = 0.038) reported increased risk for patients with higher BNIP3, whereas GSE3149 (OS: 95% CI; HR = 0.53, 0.31–0.91; *P* = 0.019), TCGA (PFS: 95% CI; HR = 0.76, 0.59–0.99; *P* = 0.038) and GSE63885 (PFS: 95% CI; HR = 0.51, 0.30–0.88; *P* = 0.015) reported the opposite results. The rest indicated no statistical relevance (Tables 3 and 4).

**Table 2:** Correlation of BNIP3 level to OS and PFS in patients with ovarian cancer with varied clinical characteristics. CI, confidence interval.

Clinical characteristics	OS			PFS		
	*N*	HR (95% CI)	*P*	*N*	HR (95% CI)	*P*
All	1656	**1.18 (1.04**–**1.34)**	**0.013**	1435	**1.26 (1.1**–**1.43)**	**0.000**
Histology						
Serous	1104	**1.23 (1.05**–**1.43)**	**0.0085**	1104	0.89 (0.75–1.05)	0.160
FIGO stage						
I + II	135	0.68 (0.31–1.49)	0.33	163	1.62 (0.84–3.13)	0.140
III + IV	1220	**1.23 (1.06**–**1.43)**	**0.0058**	1081	0.89 (0.75–1.06)	0.180
Chemotherapy						
T + P	776	1.16 (0.96–1.40)	0.13	698	0.90 (0.74–1.10)	0.300
Paclitaxel	220	**0.57 (0.36**–**0.90)**	**0.014**	229	**0.54 (0.35**–**0.84)**	**0.0053**
Gemcitabine	135	0.68 (0.45–1.02)	0.062	131	1.29 (0.86–1.92)	0.22
Topotecan	119	**0.58 (0.37**–**0.9)**	**0.013**	118	0.72 (0.46–1.13)	0.15
P53 mutation						
Mutated	506	0.88 (0.70–1.11)	0.28	483	1.19 (0.95–1.49)	0.13
Wild	94	0.71 (0.37–1.34)	0.28	84	**0.47 (0.24**–**0.93)**	**0.027**

Values in bold represent values with statistical significance.

**Table 3:** Separate analysis of correlation between BNIP3 level and OS in patients with ovarian cancer. The transcript for BNIP3 measured in each dataset was 201849_at. All studies were split by median expression of BNIP3 within each dataset. CI, confidence interval.

Dataset	No. of patients			HR (95% CI)	*P*
	Total	BNIP3 high	BNIP3 low		
TCGA	557	415	142	0.81 (0.62–1.07)	0.130
GSE9891	285	129	156	**1.46 (1.00**–**2.13)**	**0.046**
GSE26712	184	66	118	1.19 (0.83–1.71)	0.340
GSE26193	107	52	55	1.21 (0.77–1.89)	0.420
GSE63885	75	41	34	0.70 (0.43–1.14)	0.150
GSE18520	53	14	39	1.45 (0.74–2.85)	0.280
GSE3149	116	44	72	**0.53 (0.31**–**0.91)**	**0.019**
GSE30161	58	41	17	1.79 (0.79–4.05)	0.160
GSE14764	80	49	31	0.53 (0.22–1.26)	0.150

Values in bold represent values with statistical significance.

**Table 4:** Separate analysis of correlation between BNIP3 level and PFS in patients with ovarian cancer. The transcript for BNIP3 measured in each dataset was 201849_at. All studies were split by median expression of BNIP3 within each dataset. CI, confidence interval.

Dataset	No. of patients			HR (95% CI)	*P*
	Total	BNIP3 high	BNIP3 low		
TCGA	522	169	353	**0.76 (0.59**–**0.99)**	**0.038**
GSE9891	285	95	190	1.17 (0.86–1.58)	0.31
GSE26712	184	133	51	1.34 (0.93–1.93)	0.11
GSE26193	107	57	50	1.28 (0.82–1.99)	0.27
GSE65986	55	31	24	**3.53 (0.99**–**12.57)**	**0.038**
GSE63885	75	56	19	**0.51 (0.30**–**0.88)**	**0.015**
GSE30161	54	39	15	1.95 (0.95–3.99)	0.063
GSE14764	80	42	38	0.73 (0.43–1.22)	0.23

Values in bold represent values with statistical significance.

## Discussion

To evaluate the effect of BNIP3 in ovarian cancer during cisplatin treatment and whether it could work as a prognostic factor, we measured the level of BNIP3 before and after exposure to cisplatin in ovarian cancer cells, evaluated the relation of BNIP3 level to cisplatin cytotoxicity and cellular apoptosis, and examined the correlation between BNIP3 and clinical outcomes. According to our results, cisplatin‐induced apoptosis is dependent on BNIP3 level, and the depletion of BNIP3 remarkably alleviates the cytotoxic effect of cisplatin in ovarian cancer cells. These findings provide evidence of cisplatin sensitization by targeting BNIP3.

When evaluating the basic level of BNIP3 in ovarian cancer cells, we identified BNIP3 expression in all selected ovarian cancer cell lines. This finding was consistent with the former finding that BNIP3 expression was universally higher in tumor tissue compared with matched normal tissue because of hypoxia and metabolic change [23]. We also recognized big variation of basic BNIP3 level among the selected cell lines. The variation was possibly due to varied molecular background, metabolism and aggressiveness of each cell line because the basic level of BNIP3 was influenced by several factors: HIF1 level, BNIP3 promoter hypermethylation status, miRNA inhibition and hypoxia [24, 25, 26]. Cisplatin‐induced BNIP3 up‐regulation in both protein and mRNA levels was observed in ovarian cancer lines. Previous studies also reported cisplatin‐induced BNIP3 up‐regulation in lung cancer cell line A549 [27, 28]. The increase in BNIP3 could also be triggered by other drug treatments, such as rapamycin, LBH589, verticillin A and acidosis [27, 29].

In this study, BNIP3 silencing in A2780 and OVCAR4 cells led to a significant decrease in sensitivity to cisplatin, which suggested that cisplatin cytotoxicity was dependent on BNIP3 level in ovarian cancer cells. Similar results were reported in other cancer cell lines. Cisplatin cytotoxicity increased with ascending BNIP3 level in several clonal variants of human colon carcinoma cell line HT29, and up‐regulation of BNIP3 in HT29 significantly increased its cisplatin sensitivity [30]. In cisplatin‐resistant breast cancer cell line MCF‐7/R, BNIP3 expression was down‐regulated by miR‐944, and inhibition of miR‐944 restored cisplatin sensitivity of MCF7/R [26]. In lung cancer cell line A549, cisplatin‐based combination treatment triggered cell death by induction of BNIP3 [27].

BNIP3 was reported to contribute to both spontaneous and drug‐induced cell death through the apoptotic pathway. Previous studies reported that BNIP3 knockdown in the esophageal squamous cell carcinoma cell line down‐regulated spontaneous cellular apoptosis [31]. Up‐regulation of BNIP3 in colon carcinoma increased both spontaneous apoptosis and cisplatin‐induced apoptosis [30]. In this study, we observed similar results that BNIP3 silencing caused only slight reduction of spontaneous apoptosis in A2780 and a massive decrease in cisplatin‐induced apoptosis in both A2780 and OVCAR4. We found that BNIP3 silencing led to a significant decrease in cleaved PARP and cleaved caspase‐3 induced by cisplatin, which suggested that cisplatin‐induced BNIP3‐dependent apoptosis was caspase dependent. Although the underlying mechanism was not entirely clear, this result provided possible interpretation to some of the previous clinical findings. A comparative analysis and a patient‐matched analysis of 112 primary ovarian carcinoma and 63 metastatic samples reported by Nymoen *et al*. [32] showed that BNIP3 levels were significantly lower in metastatic sites than in primary ovarian cancer lesions. Also, these patients with metastasis did not respond well to cisplatin treatment, resulting in a poorer prognosis. According to our result, it was highly possible that a decrease in BNIP3 in metastatic sites alleviated cytotoxicity of cisplatin, which led to the overall failure of cisplatin‐based chemotherapy.

To unveil the mechanism of action of BNIP3 in cell death, other facets such as necrosis and autophagy should not be neglected. Although a previous study reported that BNIP3 induces programmed necrosis by an intrinsic caspase‐independent mitochondrial pathway [29], our results did not show a significant change in necrosis of ovarian cancer cells after BNIP3 modulation. One important concept that is often underappreciated is that autophagy is not necessarily responsible for promoting cell death in all cases. According to Zhu *et al*. [33], phosphorylation of BNIP3 at serines 17 and 24 initiated the binding of BNIP3 to LC3II and triggered autophagy. BNIP3‐induced autophagy counteracted apoptosis via reduction of cellular cytochrome *c* release capacity. It was demonstrated in breast cancer, pancreatic cancer, cervical cancer and other various cancers that autophagy protects against apoptosis in response to many different stimuli [34, 35, 36, 37]. In this study, we observed a significant decrease in LC3II after BNIP3 knockdown, suggesting the possible involvement of BNIP3 in autophagy. We did not explore the role of BNIP3 in autophagy further because the main consequence of BNIP3 silencing lay in reduction of cisplatin‐triggered apoptosis.

BNIP3 worked as a good prognostic indicator in pancreatic cancer [38], indicated poor prognosis in cervical cancer [34] and had inconclusive value of clinical outcome prediction in breast cancer [35]. In this study, the pooled analysis showed a correlation between high BNIP3 level with poor OS and PFS. However, considering the results of individual study and stratification analysis, the predictive value of BNIP3 in ovarian cancer should be interpreted delicately. On one hand, several individual analyses, such as the TCGA, the GSE63885 and the GSE3149 datasets, showed completely opposite results to the pooled analysis. On the other hand, the result of stratification analysis of histology, FIGO stage, chemotherapy regimen and P53 mutation status varied greatly. We assumed that such an intricate result of BNIP3 was linked to the highly inducible characteristic of BNIP3 itself and the multiple cell death pathways it was involved in.

Taken together, our results indicate that cisplatin‐induced apoptosis was dependent on BNIP3 level in ovarian cancer cell lines. Targeting BNIP3 is a promising way to restore cisplatin sensitivity.

## Conflict of interest

The authors declare no conflict of interest.

## Author contributions

XH and XY supervised the project. JJ and XH designed the present study. QZ performed the experiments. JJ and QZ collected data. XY and XH analyzed and interpreted the data. SS drafted the manuscript. SS, FY and EC prepared data and the manuscript for revision. All authors contributed to producing the manuscript and approved the final manuscript.

## Data Availability

All data generated or analyzed during this study are included in this article.
